# Retraction Note: Epidemiological characteristic of Orofacial clefts and its associated congenital anomalies: retrospective study

**DOI:** 10.1186/s12903-023-02769-7

**Published:** 2023-01-31

**Authors:** A. Impellizzeri, I. Giannantoni, A. Polimeni, E. Barbato, G. Galluccio

**Affiliations:** 1 Unit of Orthodontics, Department of Oral and Maxillofacial Sciences, “Sapienza” University of Rome, Rome, Italy; 2 Private Practice, Rome, Italy; 3 Department of Oral and Maxillo-facial Sciences, Pediatric Dentistry Unit, “Sapienza” University of Rome, Rome, Italy; 4 “Sapienza” University of Rome, Rome, Italy; 5 Department of Oral and Maxillofacial Sciences, “Sapienza” University of Rome, Rome, Italy

**Retraction Note: BMC Oral Health (2019) 19:290** 10.1186/s12903-019-0980-5

The Editor has retracted this article. After publication concerns were raised that the data presented are owned by the National Congenital Malformation Registries network of Italy [Emilia-Romagna Registry of Birth Defects (IMER) and Registro Toscano Difetti Congeniti (RTDC)]. The authors have not been able to demonstrate ownership of the data in this article. G. Galluccio and A. Impellizzeri do not agree to this retraction. I. Giannantoni, A. Polimeni and E. Barbato have not responded to correspondence from the Editor about this retraction.

